# Historical Sketch of the Work of the Board of Censors of the Medical Society of the County of Erie1Read at the seventy-fifth anniversary of the Medical Society of the County of Erie, January 14, 1896.

**Published:** 1896-07

**Authors:** Edward Storck

**Affiliations:** Buffalo, N.Y.


### HISTORICAL SKETCH OF THE WORK OF THE BOARD OF CENSORS OF THE MEDICAL SOCIETY OF THE COUNTY OF ERIE.[1]

By EDWARD STORCK, M. D., Buffalo, N. Y.

IN COMPLYING with the request of the committee of arrangements to furnish some sketches of medical events in Buffalo with which I have been identified and give an account in regard to my presidency of the society, also of my work on the board of censors, I will relate, or rather recall, some of the actions and accomplishments of the board during the twelve years that I served as its chairman. Though most of it is on record in the minutes of the society and in the files of the Buffalo Medical Journal, still it may be of some interest to our younger members on this occasion and serve for reference in the future.

I was president of the society in the year 1878, after having been a member twenty-four years. In my address to the society I made the following suggestions: "My experience as chairman of the board of health of this city, in 1872, has fully convinced me that a medical board, consisting of at least three members, two of which should be medical men, is absolutely necessary. In time

1. Read at the seventy-fifth anniversary of the Medical Society of the County of Erie, January 14, 1896.

of an epidemic, like smallpox, the people are at the mercy of the scourge with a board of laymen."

In a report made afterward to the society I submitted a bill to create a medical board of health, which was approved and a committee was appointed to secure its passage by the legislature. The common council, however, refused to endorse the bill. A former health physician, who still holds an office through his tactics, got a resolution passed against it. The provisions of the bill were similar to those embodied in our present charter.

In 1881 I was made chairman of the board of censors. At this time quackery in medicine was rampant in this county and throughout the state. Bogus medical colleges sprang up in New York and other cities, that operated under a charter obtained by the provision of an old law that gave colleges and institutions of learning or scientific and benevolent schools authority to issue diplomas. Such diplomas were sold for the same price as the Buchanan college in Philadelphia sold them, $25 apiece. County medical societies granted licenses to practise medicine to old practitioners who had never seen the inside of a medical college and who could not tell the location of the perineum. Some of the regular schools did a flourishing business as so-called doctor mills. Their aim was not so much to impart a large amount of knowledge to the students as to do a big business in turning out large graduating classes. Ignorant, illiterate persons were let loose upon the community as Doctors in Medicine and most of them settled down in this state.

The regular profession in the state was opposed to any legislation to regulate the practice of medicine. There were medical laws upon the statute books, but they were dead letters ; no one had the moral courage to enforce them.

This was the status of medical education and the practice of medicine in the state of New York in 1880. I thought there was a great field to operate upon, hence I decided to make it a study and to endeavor to effect some reforms. A few years previously I had introduced at a meeting of the state medical society, when I was a delegate, a resolution to submit to the legislature a bill to regulate the practice of medicine and medical education. The resolution was voted down. Next year I had such a bill intro duced in the legislature by one of the members from Erie county, that passed the house and senate. But when it came before Governor Hoffman, some of my orthodox colleagues in Albany prevailed upon him to veto it on the ground, as I was informed by telegram, "that the law would tacitly recognise homeopathy."

During the following session the bill was introduced again but pigeon-holed in the senate committee. In 1880 we finally succeeded in passing the medical registration law, which was signed by the governor and immediately went into force.

The first important step taken after this law passed was to effect a joint meeting of the board of censors of this society and the homeopathic county medical society. We jointly decided to execute the law, compel all legal practitioners to register their diplomas and proceed against all persons illegally practising medicine in this county. The result of our action is known to you and is on record in the reports of the board of censors. The itinerant quacks and charlatans were mercilessly dealt with as soon as they opened shop in town and got their goods well advertised in the daily papers. We didn't wish to deprive the newspapers of this profit, although some of them were quite severe on the censors for interfering with these so-called doctors or professors and their business. In handling these itinerant quacks many amusing incidents occurred, but to relate any here would make my paper too long.

The board then directed its attention to the bogus medical colleges in the state and instituted legal proceedings by applying to the attorney-general for a writ, which was granted and afterward argued before Governor Cleveland. Some of the ablest legal talent from New York and Buffalo was summoned by these schools, but the writer and his colleagues knocked them all out. The governor declared their charters null and void. Later on a regular medical school in Buffalo—Niagara University Medical College—procured a charter through a special act of the legislature. The board of censors protested and appeared before the governor. The bill was vetoed and the medical college was compelled to obtain its charter and organise under the provisions of the existing law.

The first action taken in favor of creating a state medical examining and licensing board was by the board of censors and the committee on legislation of this society. A bill to that effect was drawn and submitted to the state medical society, by which it was approved and introduced in the legislature. The bill had a hard passage. Fierce opposition was made by some of the New York colleges and after it had passed twice it was finally signed after a hard fight before Governor Hill, in June, 1890, and went into force in September, 1891.

When the state examining and licensing board went into operation my vocation as chairman of the board of censors seemed practically gone. To its chairman was left the conviction that it had been a thankless task for twelve years, but there was also the satisfaction of having done his duty and accomplished the object in view—namely, the necessary reform in the practice of medicine and medical education in the state of New York.

220 East Eagle Street.

